# The role of itaconate in host defense and inflammation

**DOI:** 10.1172/JCI148548

**Published:** 2022-01-18

**Authors:** Christian G. Peace, Luke A.J. O’Neill

**Affiliations:** School of Biochemistry and Immunology, Trinity Biomedical Sciences Institute, Trinity College Dublin, Dublin, Ireland.

## Abstract

Macrophages exposed to inflammatory stimuli including LPS undergo metabolic reprogramming to facilitate macrophage effector function. This metabolic reprogramming supports phagocytic function, cytokine release, and ROS production that are critical to protective inflammatory responses. The Krebs cycle is a central metabolic pathway within all mammalian cell types. In activated macrophages, distinct breaks in the Krebs cycle regulate macrophage effector function through the accumulation of several metabolites that were recently shown to have signaling roles in immunity. One metabolite that accumulates in macrophages because of the disturbance in the Krebs cycle is itaconate, which is derived from *cis*-aconitate by the enzyme *cis*-aconitate decarboxylase (ACOD1), encoded by immunoresponsive gene 1 (*Irg1*). This Review focuses on itaconate’s emergence as a key immunometabolite with diverse roles in immunity and inflammation. These roles include inhibition of succinate dehydrogenase (which controls levels of succinate, a metabolite with multiple roles in inflammation), inhibition of glycolysis at multiple levels (which will limit inflammation), activation of the antiinflammatory transcription factors Nrf2 and ATF3, and inhibition of the NLRP3 inflammasome. Itaconate and its derivatives have antiinflammatory effects in preclinical models of sepsis, viral infections, psoriasis, gout, ischemia/reperfusion injury, and pulmonary fibrosis, pointing to possible itaconate-based therapeutics for a range of inflammatory diseases. This intriguing metabolite continues to yield fascinating insights into the role of metabolic reprogramming in host defense and inflammation.

Macrophages, upon classical activation by the Toll-like receptor 4 (TLR4) ligand lipopolysaccharide (LPS), undergo metabolic reprogramming to meet newly required bioenergetic and biosynthetic demands that regulate macrophage effector function. Generally, anabolic pathways that fuel biosynthesis, such as aerobic glycolysis (termed the “Warburg effect”), are highly upregulated, whereas catabolic pathways such as oxidative phosphorylation (OXPHOS) become downregulated (1). In LPS-activated macrophages, the Krebs cycle (also known as the citric acid or tricarboxylic acid cycle) and OXPHOS are initially upregulated, followed by the nitric oxide–mediated disruption of the Krebs cycle and respiratory chain (2–7), resulting in the accumulation of immunometabolites such as succinate, fumarate, and itaconate, which exhibit a wide range immunoregulatory functions (8–11).

In 2011, itaconate was shown to be a mammalian metabolite produced in large quantities in LPS-activated macrophages (12) and present in the lungs of mice infected with *Mycobacterium tuberculosis* (13). Michelucci et al. then identified *cis*-aconitate decarboxylase (ACOD1), encoded by immunoresponsive gene 1 (*Irg1*), as the enzyme responsible for the decarboxylation of the Krebs cycle intermediate *cis*-aconitate in macrophages (14). ACOD1 had been identified as an LPS-inducible cDNA with no known function in 1995 (15). To date, macrophages and myeloid cells are the only cells that have been found to produce itaconate in response to M1-polarizing conditions (e.g., LPS exposure). Since its identification, itaconate has emerged as a mitochondrial metabolite with antimicrobial but also immunomodulatory properties. In this Review, we discuss these properties and speculate on the therapeutic potential of itaconate in inflammatory diseases.

Structurally, itaconate is a five-carbon dicarboxylic acid with an α,β-unsaturated alkene (Figure 1 and ref. 16). It is structurally and chemically similar to other metabolites such as phosphoenolpyruvate, succinate, malonate, and fumarate. Such similarities have informed studies of its antimicrobial and immunoregulatory effects. Esterified derivatives of itaconate, 4-octyl itaconate (OI) and dimethyl itaconate (DI) (Figure 1), are commonly used to mimic its biological effects in vitro and in vivo because of their high membrane permeability, as it was unclear until recently whether underivatized itaconate is able to be taken up by macrophages (17). However, increased electrophilicity of derivatives, or other chemical differences, may reveal effects that are not attributable to endogenously produced itaconate. The use of derivatives is an important area for the field and will be discussed in more detail; it may point the way to novel therapeutics based on itaconate.

## Roles for itaconate in host-pathogen interactions

**Figure 2:**
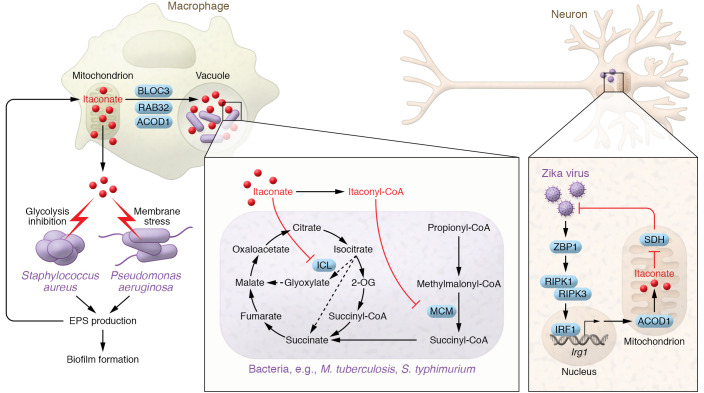
The effect of itaconate on bacteria and viruses. Upon exposure to pathogens, *Irg1* is induced, mediating the production of Krebs cycle–derived itaconate within mitochondria (7). In neurons, *Irg1* is induced through ZBP1, RIPK1/3, and IRF1 to restrict Zika viral replication through inhibition of SDH (49). Itaconate is also produced to inhibit the growth of bacteria such as *Mycobacterium tuberculosis* and *Salmonella* (28, 33). A notable antimicrobial mechanism is the inhibition of bacterial isocitrate lyase (ICL), which blocks the glyoxylate shunt that is required for optimal growth and pathogenicity (19, 21). The breakdown product of itaconate, itaconyl-CoA, is also an inhibitor of methylmalonyl-CoA mutase (MCM) in bacteria (e.g., *M. tuberculosis*), thereby blocking propionyl-CoA–dependent bacterial growth (28). Recently, itaconate was shown to be delivered to *Salmonella*-containing vacuoles, which limits bacterial growth. Mechanistically, this was found to occur through the Rab32 GTPase and its exchange factor, BLOC3, which interact with ACOD1 to target itaconate to bacteria contained within vacuoles (33). Although itaconate is antimicrobial, some pathogens, such as *Pseudomonas aeruginosa* and *Staphylococcus aureus*, have developed ways to exploit itaconate to fuel biofilm formation (43, 47). Itaconate causes membrane stress of *P*. *aeruginosa* and inhibits aldolase (and glycolysis) in *S*. *aureus*, both of which lead to the production of extracellular polysaccharides (EPSs), which further induce *Irg1* expression and itaconate synthesis in a positive-feedback manner. EPSs also facilitate the formation of bacterial biofilms, promoting growth and survival of *P*. *aeruginosa* and *S*. *aureus*.

Since the identification of itaconate as a mammalian metabolite, many studies have characterized roles for itaconate in host-pathogen interactions (Figure 2). Key inducers of the IRG1/itaconate axis in myeloid cells, such as exposure to LPS and acute iron deprivation, occur during infection, indicating an important role for itaconate in infection (18). A notable antimicrobial mechanism of itaconate is its activity as a bacterial isocitrate lyase (ICL) inhibitor (19–21). ICL is a bacterial enzyme required for the glyoxylate shunt during bacterial infection (22–25). As such, inhibition of ICL by itaconate limits growth of pathogens that depend on ICL activity such as *Pseudomonas indigofera* (19, 21, 26, 27). Recently, a breakdown product of itaconate metabolism, itaconyl-CoA, was also shown to limit the growth of *M. tuberculosis* (28). During *M. tuberculosis* infection, cholesterol-derived propionyl-CoA metabolism is required for optimal pathogenicity (29). Itaconyl-CoA, which had previously been shown to inhibit human 5-deoxyadenosylcobalaman-dependent methylmalonyl-CoA mutase (hMCM; ref. 30), was also found to inhibit propionate-dependent *M. tuberculosis* growth (28), which requires MCM activity (31). Additionally, itaconate prevents *M. tuberculosis–*associated immunopathology by reducing macrophage chemokine production and inhibiting neutrophil recruitment to the lungs of *M. tuberculosis–*infected mice (32).

Recently, itaconate’s role as a novel host defense mechanism was demonstrated when the Rab32 GTPase was shown to interact with ACOD1 to facilitate the delivery of itaconate into *Salmonella*-containing vacuoles (SCVs) (33). Interestingly, *Salmonella typhimurium* is able to evade this antimicrobial mechanism through the expression of the secretion effectors SopD2 and GtgE (34, 35), suggesting potential evolutionary selection of itaconate-resistant strains of *Salmonella*. *Salmonella* deficient in SopD2 and GtgE replicate poorly specifically in cells of hematopoietic origin, while in numerous other cell types SopD2 and GtgE expression did not affect replication, suggesting that they are acting on a pathway unique to hematopoietic cells. Next, a proteomic screen for Rab32-interacting proteins revealed the interaction of ACOD1 and Rab32, prompting examination of a role for itaconate. The delivery of itaconate into SCVs was demonstrated using a strain of *Salmonella* expressing an itaconate biosensor, showing that bacteria contained within a vacuole were exposed to higher concentrations of itaconate compared with cytoplasmic bacteria, as there was a greater level of reporter expression within the vacuole. Bone marrow–derived macrophages (BMDMs) from mice lacking Rab32 and BLOC3 (the exchange factor required for Rab32 function) produced normal levels of itaconate but lacked expression of the itaconate reporter in SCVs, indicating Rab32’s essential role in delivering itaconate into SCVs. Importantly, deficiency of BLOC3 and ACOD1 resulted in increased replication of *S*. *typhimurium*, and both BLOC3 and ACOD1 were shown to be required for the increased pathogenicity of SopD2/GtgE-expressing *S*. *typhimurium*. Together, these studies highlight itaconate’s antimicrobial properties.

Interestingly, while itaconate is a natural antimicrobial molecule, some pathogens have evolved elegant mechanisms to overcome itaconate during infection. For example, some bacteria are able to break down and metabolize itaconate to pyruvate and acetyl-CoA. Itaconate degradation has been characterized in bacteria for years; however, the enzymes responsible and their role during infection remained unclear until recently when *Yersinia pestis* and *Pseudomonas aeruginosa* were shown to contain several genes encoding the enzymes itaconate CoA transferase, itaconyl-CoA hydratase, and (*S*)-citramalyl-CoA lyase, which are required for itaconate catabolism, allowing increased survival within macrophages (36–39). Similarly, *M. tuberculosis* expresses the bifunctional enzyme β-hydroxyacyl-CoA lyase, which is required for itaconate dissimilation and leucine catabolism, and in turn this promotes *M. tuberculosis* pathogenicity (40). It is also worth noting that there appears to be an interesting relationship between itaconate metabolism and leucine metabolism, as enzymes involved in the catabolism of leucine (e.g., BCAT1) are required for IRG1 expression and itaconate production in macrophages (4, 41).

### P.

*aeruginosa* and *Staphylococcus aureus* are two striking examples of pathogens able to exploit host-derived itaconate to fuel biofilm formation, resulting in increased pathogenicity and survival (42, 43). Due to its electrophilicity, itaconate induces a prosurvival membrane stress response in *Pseudomonas* (44, 45). Under conditions of membrane stress, *P*. *aeruginosa* favor extracellular polysaccharide (EPS) synthesis over LPS biosynthesis, which in turn was found to further drive metabolic reprogramming of macrophages, augmenting itaconate synthesis in a positive-feedback loop (43). EPS production also led to increased biofilm formation, providing a survival niche for *P*. *aeruginosa* in the lung (46). In a similar manner, itaconate has also recently been shown to be an important factor able to drive biofilm formation during *S*. *aureus* infection (47). The induction of itaconate by *S*. *aureus* leads to itaconate-mediated inhibition of staphylococcal aldolase, resulting in the redirection of fructose-6-phosphate to glucosamine-6-phosphate to fuel EPS biosynthesis and subsequent biofilm formation. These studies, therefore, further emphasize the role of itaconate as an antibacterial metabolite that some bacteria can subvert.

Interestingly, itaconate may also have antiviral properties. Daniels et al. (48) demonstrated that Zika virus–infected neurons upregulate *Irg1* expression in a ZBP1/RIPK3/IRF1-dependent manner to restrict Zika viral replication. In mice infected with Zika virus, *Irg1* deficiency led to increased viral replication and decreased survival, confirming the importance of the pathway in vivo during viral infections. Both OI and dimethyl malonate, an inhibitor of succinate dehydrogenase (SDH), had similar effects in restricting viral replication, indicating that inhibition of SDH by itaconate may potentially be a host antiviral defense mechanism. This study exemplifies a robust approach whereby a derivative (in the case of OI) has an effect (antiviral) that is consistent with an effect on *Irg1*-deficient mice (enhanced viral replication), suggesting that endogenous itaconate is antiviral.

Similarly, it has been found that OI and the fumarate derivate dimethyl fumarate (DMF) are able to suppress SARS-CoV-2 replication (49). OI and DMF are both activators of Nrf2, an antioxidant and antiinflammatory transcription factor that senses oxidative and electrophilic stress. Initially, RNA-Seq analysis found Nrf2-dependent genes (e.g., *NQO1*, *GCLM*, and *HMOX1*) to be downregulated in COVID-19 patients, suggesting that impairment of the Nrf2 pathway may be a potential host evasion mechanism by SARS-CoV-2. In support of this, Nrf2 activation by OI or DMF, and knockdown of KEAP1 (a negative regulator of Nrf2), were shown to inhibit SARS-CoV-2 replication as well as the replication of other pathogenic viruses including herpes simplex virus 1 (HSV-1), vaccinia virus, and Zika virus. Additionally, this study found that expression of inflammatory genes such as *IFNB1*, *CXCL10*, *TNF*, *IL1B*, and *CCL5*, which are key drivers of pathology, is blocked by OI and DMF upon SARS-CoV-2 and HSV-1 infection, indicating that targeting Nrf2 in COVID-19 or other viral infections may have therapeutic potential as both an antiviral and antiinflammatory strategy. Recently, activation of Nrf2 by itaconate has also been shown to be therapeutically effective in treating ocular bacterial infection (50).

Supporting the idea that itaconate may have a role in host defense, several single-nucleotide polymorphisms (SNPs) have recently been identified in *IRG1*, resulting in increased enzyme activity and itaconate production (51). Although the functional relevance of these SNPs has not been fully characterized, it is conceivable that increased itaconate may lead to improved host protection in the context of infection. The high prevalence of genetic variants in populations in Africa possibly indicates evolutionary importance of the *IRG1*/itaconate pathway, as these variants may have arisen due to selective pressure from infection.

## Immunomodulatory properties of itaconate

**Figure 3:**
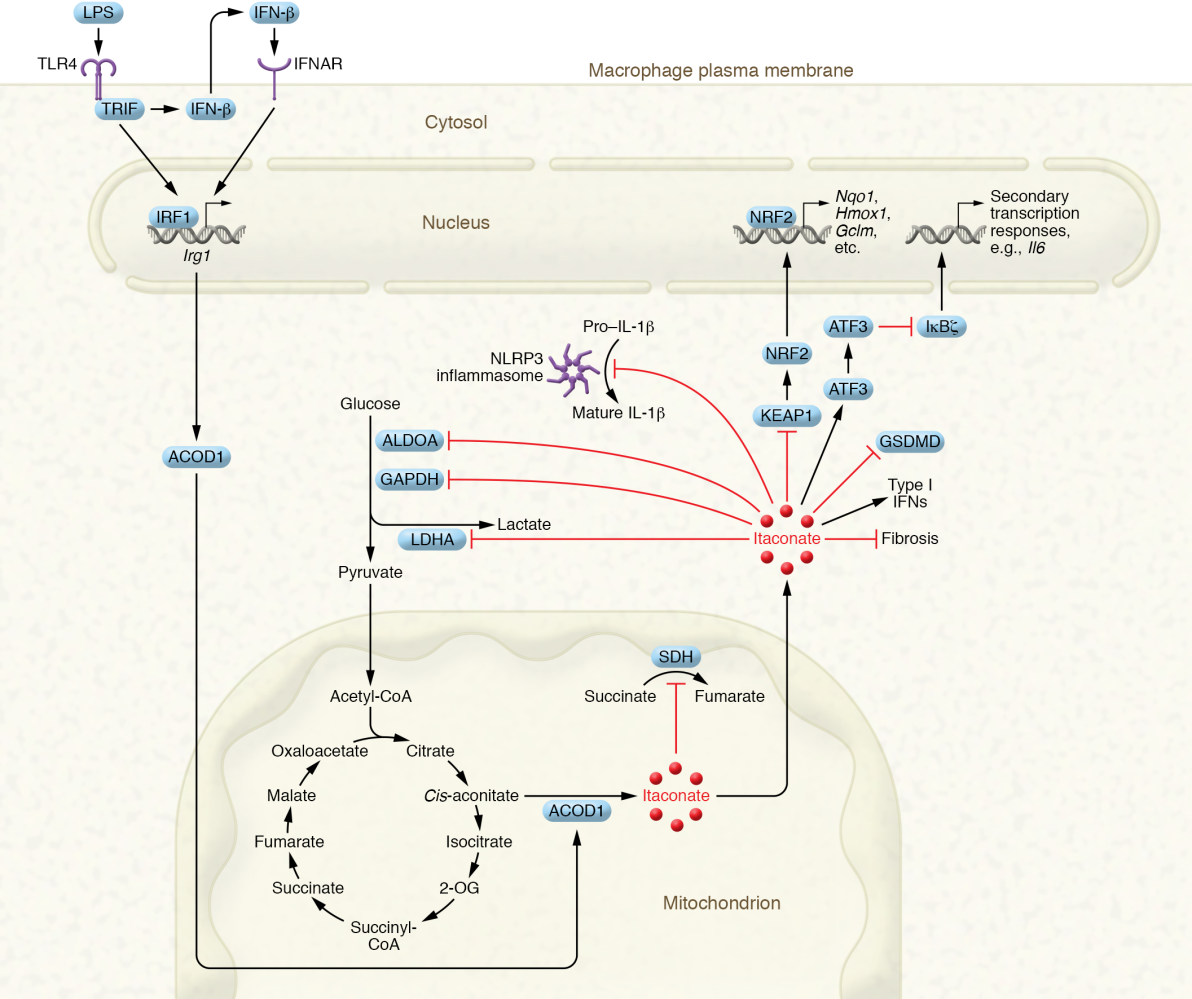
The immunoregulatory properties of itaconate. *Irg1* is induced by LPS in a TRIF-dependent manner, leading to itaconate production (7, 73). Taking evidence from itaconate derivatives or from *Irg1*-deficient macrophages, several targets of itaconate, including succinate dehydrogenase (SDH), have been identified, which prevent the oxidation of succinate to fumarate and decrease mtROS production (53, 54). Itaconate also exits the mitochondria, where it has numerous antiinflammatory effects (11). A key mechanism of itaconate is the modification of thiol-reactive cysteines, many of which have been identified by proteomic screens. Targets include the glycolytic enzymes aldolase A (ALDOA; ref. 76), lactate dehydrogenase A (LDHA; ref. 11), glyceraldehyde-3-phosphate dehydrogenase (GAPDH; ref. 78), and the NLRP3 inflammasome, which will prevent processing of IL-1β, IL-18, and GSDMD. Nrf2 and activating transcription factor 3 (ATF3) have also been identified as possible targets (11, 69). Additionally, in alveolar macrophages, itaconate has been shown to repress the severity of lung fibrosis (106). Finally, itaconate has also been reported to boost type I IFN signaling by an undetermined mechanism (17).

Beyond its role as an antimicrobial metabolite, itaconate has garnered the attention of immunologists because of its immunomodulatory properties (Figure 3), which are still being explored. Below, we review evidence for itaconate’s function in innate immunomodulation, focusing on its role as an SDH inhibitor, an Nrf2 activator and cysteine modifier, and a regulator of the ATF3/IκBζ axis, glycolysis, type I IFNs, and the NLRP3 inflammasome. We discuss the effects of derivatives of itaconate relative to the parent molecule, where there are some overlaps but also some important differences.

### Itaconate as an SDH inhibitor.

SDH (also known as complex II of the electron transport chain) catalyzes the oxidation of succinate to fumarate in LPS-activated macrophages and supports the metabolic reprogramming that drives a proinflammatory phenotype (52). In 2016, the first immunoregulatory role of itaconate was shown to be its inhibition of SDH (53, 54). While it had been known for decades that itaconate was a competitive inhibitor of SDH owing to its structural similarity to succinate (55–57), potential physiological roles of itaconate as an endogenous SDH inhibitor had been unexplored. Inhibition of SDH prevents the oxidation of succinate to fumarate, thereby preventing the generation of complex I–driven mitochondrial reactive oxygen species (mtROS) (52, 58). The inhibition of mtROS supports prolyl hydroxylase activity to suppress stabilization of hypoxia-inducible factor (HIF) and thereby block the transcription of proinflammatory IL-1β (59–61).

The cell-permeable itaconate derivative DI inhibited the expression of numerous proinflammatory genes, such as those encoding IL-1β and IL-18, thereby linking itaconate to modulation of macrophage function (53). Furthermore, *Irg1^–/–^* BMDMs were reported to be unable to accumulate succinate after exposure to LPS (indicating catalytically active SDH), and *Irg1* deficiency also promoted HIF stabilization. In response to inflammatory stimuli, *Irg1* deficiency increased nitric oxide production and the release of proinflammatory cytokines including IL-6, IL-1β, IL-18, and IL-12p70 relative to *Irg1*-intact BMDMs, but TNF remained unaffected, pointing to specificity of this pathway.

Inhibition of SDH by itaconate has been shown to be an important regulator of immune tolerance and trained immunity (62). Upon stimulation of CD14^+^ human monocytes by LPS, itaconate was shown to induce innate immune tolerance (or immunoparalysis), whereas the fungal cell wall component β-glucan was able to reverse this effect through the transcriptional downregulation of *IRG1* and epigenetic upregulation of SDH. To verify the roles of *IRG1* and SDH in the regulation of immune tolerance and trained immunity, a large human cohort was used to identify SNPs that affected the expression of *IRG1* and SDH subunits. Strikingly, these SNPs were found to affect IL-6, lactate production, and TNF expression, underscoring the physiological importance of this pathway in humans (62).

### Itaconate as an Nrf2 activator and cysteine modifier.

Nrf2 is a key antioxidant transcription factor that is able to suppress proinflammatory responses in macrophages (63). It is regulated posttranslationally by Kelch-like ECH-associated protein 1 (KEAP1), which mediates the degradation of Nrf2 (64). Upon exposure to oxidative or electrophilic stress, KEAP1 dissociates from Nrf2, allowing its translocation into the nucleus and the induction of antioxidant Nrf2-target genes such as *Hmox1* and *Nqo1* (65–68). Antioxidant responses decrease cellular ROS, which suppresses HIF-1α. Nrf2 has also been shown to actively repress proinflammatory gene transcription in macrophages (63, 69), prompting much interest in it as a target in inflammation.

Evidence from *Irg1^–/–^* macrophages has indicated a role in the activation of Nrf2 (69). As an α,β-unsaturated dicarboxylic acid, itaconate is mildly electrophilic, allowing it to act as a Michael acceptor to modify cysteine residues in a process termed “2,3-dicarboxypropylation” (also known as “itaconation”) (11). As such, itaconate and its more electrophilic esterified derivatives, DI and OI, have been found to activate Nrf2 (11, 69, 70). Nrf2 activation by OI was shown to be dependent on the alkylation of KEAP1 cysteine residues, thereby preventing KEAP1-mediated degradation of Nrf2 (11). Additionally, activation of Nrf2 was found to be a predominant mechanism of OI-mediated inhibition of IL-1β, as inhibition of IL-1β by OI required both the critical thiol-reactive KEAP1 cysteines and the presence of Nrf2 (11). Although the modification of cysteines on KEAP1 was shown with OI, recently the identification of KEAP1 cysteine modifications has been found with underivatized itaconate, further supporting this pathway (71). OI and DI did not inhibit TNF production, p65 phosphorylation, or primary transcriptional responses to LPS, which also indicates specificity toward Nrf2 (11, 69). Recently, contradictory findings suggest that endogenous itaconate does not strongly activate Nrf2-driven responses in the context of LPS and that itaconate and OI do not require Nrf2 to exert antiinflammatory activities (72). This was, however, in the context of using particulate matter as the stimulus. Currently, most data would suggest that itaconate exerts electrophilic properties that activate Nrf2 and ATF3 in the context of LPS stimulation, while pretreatment in resting macrophages with underivatized itaconate has been shown to not strongly activate electrophilic stress signatures (17, 73).

Recently, Nrf2 has been shown to inhibit stimulator of interferon genes (STING) gene expression, leading to impaired type I IFN production in response to STING agonists (74). As such, activation of Nrf2 by OI was found to represses STING and downstream responses, suggesting that this could represent a partial mechanism by which OI and DI reduce type I IFN signaling (discussed in greater detail below). The link between Nrf2 and type I IFN signaling was supported by the observation that KEAP1 knockdown also decreased STING expression. cGAMP (a STING agonist) was also unable to induce IRF3 phosphorylation (an indicator of STING activation) in the KEAP1-knockdown group (74). In further support, Nrf2 knockdown resulted in increased STING expression, and upon activation of STING, Nrf2 deficiency increased phosphorylation of TBK1 (a downstream mediator of STING activation), STAT1 phosphorylation, *IFIT1* expression, *ISG15* expression, and *IFNB1* gene expression (74).

Numerous screens in search of roles of itaconate as a cysteine modifier have identified many cysteine residues potentially modified by itaconate. These screens have been carried out in LPS-activated macrophages (11) and in LPS-activated tolerized macrophages (73), as well as with itaconate tool compounds (e.g., itaconate alkyne probes) and chemoproteomic profiling methods (75, 76), identifying many potential targets and revealing potential novel mechanisms of action of itaconate (11, 53, 69, 75, 77). While many modifications are not functionally validated, cysteine mutants such as those used by Mills et al. (KEAP1 mutants; ref. 11) and Liao et al. (GAPDH mutants; ref. 78) are able to demonstrate which cysteine modifications may have functional importance in the context of OI. The evidence that endogenous itaconate can give rise to functional consequences by modifying cysteines remains circumstantial.

*Itaconate as a regulator of the ATF3/IκBζaxis*. IκBζ (encoded by *Nfkbiz*) and activating transcription factor 3 (ATF3) signal in a proinflammatory axis that is independent of Nrf2 (69). Electrophilic stress caused by itaconate and its derivatives has also been shown to inhibit IκBζ via the upregulation of genes associated with the unfolded protein response, most notably that encoding the antiinflammatory transcription factor ATF3 (69). Ultimately, the upregulation of ATF3 by DI was found to inhibit IκBζ, resulting in decreased production of proinflammatory IL-6. The observed downregulation of type I IFN–related genes by DI, which was shown to be Nrf2 independent, could be through ATF3 activation, as ATF3 has been identified as a key regulator of type I IFN responses (79). Since DI failed to inhibit primary transcriptional responses to LPS at early time points and did not affect TNF release, it was reasoned that downregulation of secondary transcriptional response genes such as those encoding IL-6 and IL-12 may be a more physiologically important mechanism of action of DI. Conceptually, the slow buildup of endogenous itaconate within LPS-activated macrophages supports this idea.

Furthermore, *Irg1^–/–^* tolerized macrophages restimulated with LPS were found to have lower levels of ATF3 and increased IκBζ, consistent with endogenous itaconate regulating this proinflammatory process.

### Itaconate as a glycolytic inhibitor.

The glycolytic metabolic program that is initiated upon macrophage exposure to LPS is required for optimal inflammatory responses (80). Several recent studies have suggested that the antiinflammatory functions of itaconate and derivatives may be through regulation of aerobic glycolysis (76, 78). Alkylated cysteine residues have been identified in several enzymes involved in glycolysis, including aldolase A (ALDOA), glyceraldehyde-3-phosphate dehydrogenase (GAPDH), and lactate dehydrogenase A (LDHA) (11, 76, 78). Qin et al. (76) reported that itaconate decreases ALDOA enzyme activity, glucose consumption, and lactate production and that *Irg1^–/–^* macrophages have increased ALDOA activity. Liao et al. (78) showed that OI alkylates cysteine 22 (C-22) of GAPDH. Functionally, it was found that OI inhibited GAPDH enzyme activity, lactate production, and extracellular acidification rate (ECAR), which is consistent with glycolytic inhibition. The effects of OI on the expression of IL-1β, TNF, and iNOS, as well as NF-κB nuclear translocation, closely reflected effects by the highly specific GAPDH inhibitor heptelidic acid, suggesting that GAPDH may be a key target of OI (78). Confirming the physiological importance of C-22 of GAPDH, cells expressing mutant GAPDH with C-22 substituted by alanine were generated and shown to produce less IL-1β, indicating the functional requirement of C-22 in sustaining GAPDH activity (78). Exploring whether endogenous itaconate had a role in regulating GAPDH, the authors also found that *Irg1^–/–^* BMDMs had increased GAPDH enzyme activity, increased lactate production, and increased ECAR (78).

Additionally, itaconate has also been shown to be an inhibitor of fructose-6-phosphate 2-kinase, acting in a similar manner to the itaconate analog phosphoenolpyruvate (a glycolytic intermediate) (81), thereby decreasing fructose-2,6-biphosphate synthesis (82). Since fructose-2,6-biphosphate activates phosphofructokinase activity, it is conceivable that a decrease in levels of fructose-2,6-biphosphate by itaconate may contribute to an inhibition of glycolysis in LPS-activated macrophages.

Notably, GAPDH has also been reported to be a target of dimethyl fumarate (DMF) (83), and screens for cysteine succination in fumarate hydratase–deficient cells (which accumulate endogenous fumarate) have found that both GAPDH and LDHA are succinated by fumarate as well (84). It would therefore appear that both itaconate and fumarate regulate glycolysis in inflammatory macrophages, pointing to its importance in macrophage function.

### Regulation of type I IFNs by itaconate.

Recently, Swain et al. (17) demonstrated the importance of confirming mechanistic studies carried out with derivatives using underivatized itaconate and *Irg1^–/–^* macrophages. Although it was previously unclear whether underivatized itaconate was cell permeable, it was found that at high concentrations exogenous itaconate can be taken up by BMDMs, which was supported by an increase in succinate levels. DI and OI failed to increase intracellular levels of itaconate and were unable to induce succinate accumulation in *Irg1*-deficient macrophages, indicating that immunomodulatory effects of derivatives might be independent of SDH inhibition (17). Other reports suggest that OI directly generates intracellular itaconate (11, 77, 78); however, it is important to note that these studies were performed in LPS-activated macrophages, that a large amount of OI is not metabolized to itaconate, and that exogenous itaconate treatment as used by Swain et al. (17) is able to produce much greater levels of endogenous itaconate. Additionally, DI and OI were found to significantly deplete glutathione (GSH) levels, while exogenous itaconate did not (17), indicating increased electrophilicity of the derivatives and underscoring the need to verify physiological roles of itaconate in *Irg1^–/–^* mice or with exogenous itaconate.

Notably, this study reported a key difference between itaconate and its derivatives in the regulation of type I IFNs (17). Type I IFNs secreted from activated macrophages have numerous roles in defense against infections (85). Itaconate was initially thought to inhibit type I IFN responses, as studies using OI and DI found a strong decrease in type I IFN–related genes in LPS-activated macrophages (11, 69). However, Swain et al. (17) found that underivatized itaconate and esterified derivatives differ in their regulation of type I IFNs. Strikingly, *Irg1^–/–^* BMDMs were found to have an attenuated induction of type I IFNs in response to LPS, whereas treatment with underivatized itaconate rescued the induction of type I IFN–related genes, demonstrating that itaconate is involved in the upregulation of type I IFNs in macrophages. Mechanistically, it is currently unclear precisely how the derivatives reduce type I IFNs or how itaconate augments type I IFN production. Although some evidence points to the involvement of Nrf2 (11, 74), RNA-Seq analysis found that the inhibition of type I IFN–related genes by DI is independent of Nrf2 (69).

### Itaconate as an NLRP3 inflammasome inhibitor.

A key emerging role for itaconate is its suppression of the NLRP3 inflammasome, an innate immune mechanism that promotes proinflammatory cytokine secretion after sensing a priming signal such as microbial products (e.g., LPS) and a second activating signal such as cellular damage (e.g., ATP) (86). Lampropoulou et al. (53) had initially found an increase of IL-1β and IL-18 release in *Irg1^–/–^* BMDMs stimulated with LPS and ATP (53); however, mechanistically it remained unclear precisely how itaconate inhibited inflammasome activation and IL-1β/IL-18 release. Partly, this could be attributed to a reduction of pro–IL-1β transcriptionally through SDH inhibition or Nrf2 activation (11, 53). However, these effects of itaconate would not be able to account for any potential effects of itaconate on the ATP-mediated signal (hereafter referred to as signal 2), which has been shown to actually require Nrf2 (87). Additionally, Swain et al. (17) demonstrated that exogenous itaconate specifically decreased IL-1β release whereas there was no effect on transcription, pointing to direct modulation of NLRP3 activity and the possible targeting of signal 2 for NLRP3 activation.

Two recent studies have described detailed mechanisms by which itaconate may inhibit NLRP3 activation (73, 77). Hooftman et al. (77) attributed the observed effects on suppression of NLRP3 activation by itaconate to the alkylation of cysteine 548 (C-548) on NLRP3 (77). OI was added after the initial LPS stimulation to examine effects of itaconate specifically on signal 2. To exclude potential effects of OI on LPS-mediated NLRP3 signals (hereafter referred to as signal 1) through SDH or Nrf2, treatment after LPS was shown to have no effect on pro–IL-1β expression. OI inhibited lactate dehydrogenase (LDH) release, IL-18 release, ASC speck formation, and gasdermin D (GSDMD) and IL-1β processing, all of which are indicators of NLRP3 activation. OI was found to have no effect on the NLRC4 or AIM2 inflammasomes. Additionally, exogenous itaconate and *Irg1*-deficient BMDMs confirmed the effects observed using OI, supporting that this is an endogenous pathway. Finally, in a system whereby the murine inflammasome was reconstituted in HEK293 cells, overexpression of *Irg1* suppressed IL-1β release. Dicarboxypropylation of C-548 of NLRP3 might be responsible for the disruption of the NLRP3/NEK7 complex observed with OI.

Bambouskova et al. (73) described a mechanism whereby itaconate and iNOS synergize to tolerize late NLRP3 inflammasome activation using a model of longer LPS priming (12–24 hours) followed by signal 2 (the late inflammasome). Both caspase-1 and IL-1β processing was present in late inflammasome–induced *Irg1^–/–^* BMDMs, while wild-type BMDMs were unable to process caspase-1 and IL-1β at the later time point because of tolerance. In contrast to Hooftman et al., this study suggests that itaconate is acting downstream of ASC speck formation, as wild-type BMDMs activated classically had levels of ASC speck formation similar to those in tolerized macrophages (73). Additionally, GSDMD cleavage and pyroptosis were restored upon late inflammasome activation in *Irg1^–/–^* BMDMs, whereas these were completely abrogated in wild-type BMDMs. Interestingly, the addition of cell-permeable glutathione ethyl ester to wild-type BMDMs rescued IL-1β secretion upon activation of the late inflammasome, suggesting that electrophilic stress or thiol reactivity may be responsible for inflammasome tolerance induced by itaconate. To further ascertain the mechanism, a proteomic screen was performed, which identified numerous potential targets for itaconate during late inflammasome activation. Most notably, cysteine 77 of GSDMD was identified as a possible target of itaconate. Although GSDMD is traditionally understood to be downstream of caspase-1, caspase-1 activity upon late inflammasome activation was largely GSDMD dependent, suggesting that GSDMD may be required for late inflammasome activation and the processing of caspase-1. Therefore, it is reasonable to consider the alkylation of GSDMD by itaconate as a potential mechanism of NLRP3 inhibition by itaconate.

## Therapeutic implications

The many immunoregulatory roles described for itaconate have uncovered its potential as a therapeutic for numerous diseases. In support of this, many of the studies discussed above have included relevant murine disease models demonstrating therapeutic effects of itaconate (summarized in Table 1). Therapeutically, lessons from natural properties of itaconate may also inform us of novel drug targets that may be worth pursuing. For instance, there is currently much interest in modulating NLRP3, Nrf2, and GAPDH in inflammatory diseases. While itaconate derivatives continue to show success in disease models, alternative approaches could aim to increase endogenous itaconate through the upregulation of *Irg1* or through the development of enzymatic activators of ACOD1.

### Itaconate in ischemia/reperfusion injury.

In addition to the proinflammatory role of SDH in macrophages, in ischemia/reperfusion injury (IRI), SDH drives mtROS production, promoting pathology. Thus, SDH inhibition by dimethyl malonate (DMM) has shown potential in treating IRI (88). Due to the shared ability of itaconate and derivatives to inhibit SDH activity, they may also have therapeutic potential in treating IRI in a similar manner to malonate derivatives (89). As such, administration of both DI and itaconate have been shown to improve outcome in cardiac and cerebral ischemia/reperfusion murine models (53, 90), suggesting that targeting SDH by itaconate could be therapeutically viable.

### Itaconate in sepsis.

Sepsis results from pathological systemic inflammation and catabolism and leads to damage in multiple organs. In murine models of sepsis, OI has been shown to increase survival and prevent LPS-induced lethality (11, 78). In vivo, OI had been shown to decrease proinflammatory cytokine and lactate production in response to LPS challenge (11, 78). Mechanistically, it is difficult to pinpoint particular targets of OI required for its prosurvival effects in sepsis, although it is likely a combination of numerous targets such as Nrf2 and GAPDH (91). As SDH inhibition by DMM has been shown to reduce inflammatory cytokines in vivo (52), the inhibition of SDH by itaconate could contribute to its ability to prevent lethality in sepsis as well.

### Itaconate in psoriasis.

In epithelial cells, the induction of IκBζ has been reported to be driven in an IL-17–dependent manner (92–94). Furthermore, polymorphisms in *Nfkbiz* have been implicated in driving psoriasis (95). Therefore, regulation of IκBζ by DI may be used to treat IL-17–driven diseases including psoriasis. To investigate this potential further, DI was studied in a murine model of psoriasis whereby imiquimod (IMQ) (a TLR7/8 agonist) was applied to ear tissue (69). In the model, DI was able to suppress IκBζ expression in keratinocytes as well as common IκBζ targets such as *Defb4* (encoding β-defensin-2), *S100a9* and *S100a7a* (encoding S100 calcium-binding proteins implicated in inflammation), and *Lcn2* (encoding lipocalin-2). Additionally, pathology was prevented by DI, as the ear thickness of IMQ-treated mice remained comparable to that of naive mice. *Irg1* deficiency has also been shown to result in increased IL-17A–producing T cells in the ears of IMQ-treated mice (73), likely as a result of increased IL-1β release (96, 97). These models suggest that DI or other itaconate-based therapeutics may be promising in the treatment of IL-17–associated pathologies through the ATF3-mediated downregulation of IκBζ expression.

### Itaconate in NLRP3-driven diseases.

NLRP3 has been implicated in a range of inflammatory, cardiovascular, and neurological diseases (97–103). As such, it is an increasingly exciting and attractive therapeutic target. The identification of NLRP3 as a new target of itaconate may therefore yield potential therapeutics for NLRP3-associated diseases. Both underivatized itaconate and derivatives have shown efficacy in treating NLRP3-driven disease models in vivo. Notably, sodium itaconate has been shown to reduce serum IL-1β following an intraperitoneal LPS challenge. In mice, intraperitoneal administration of monosodium urate (MSU) crystals (the driver of gout) activates the NLRP3 inflammasome (104); however, coadministration of OI with MSU crystals was able to reduce levels of IL-1β, IL-6 (downstream of IL-1β signaling), and neutrophil recruitment in peritoneal lavage fluid (77). OI was also used in peripheral blood mononuclear cells (PBMCs) isolated from patients with cryopyrin-associated periodic syndrome (CAPS), a class of diseases characterized by mutations in *NLRP3* leading to spontaneous inflammasome activation (105). Gratifyingly, OI reduced IL-1β release in PBMCs from CAPS patients treated with LPS (77), demonstrating that itaconate-based therapeutics could be effective against NLRP3-associated diseases in humans.

### Itaconate in pulmonary fibrosis.

Recently, itaconate has been identified as a key regulator of pulmonary fibrosis (106). Its role may be clinically relevant, as patients with idiopathic pulmonary fibrosis (IPF) were found to have decreased *IRG1* expression in alveolar macrophages (AMs) and low concentrations of itaconate in bronchoalveolar lavage (BAL) fluid. To examine the physiological role of itaconate in pulmonary fibrosis, the bleomycin murine model was used, which involves an inflammatory phase at day 7, a peak fibrotic phase at day 21, and a late fibrotic phase at day 42. In this model, *Irg1* and itaconate were found to be highly induced, suggesting potential physiological relevance. At later stages of fibrosis, loss of *Irg1* resulted in increased levels of AMs and neutrophils in BAL fluid. Furthermore, lungs of *Irg1^–/–^* mice had worsened dynamic resistance, elastance, and compliance (markers of disease severity). The enhanced fibrosis at the late stage was also marked by increased lung hydroxyproline, increased Ashcroft scoring (a method of estimating severity of fibrosis; ref. 107), and higher levels of lung superoxide in *Irg1^–/–^* mice. Remarkably, the adoptive transfer of wild-type monocyte-derived AMs into *Irg1^–/–^* mice at day 7 was able to improve the severity of pulmonary fibrosis along with decreased Ashcroft scoring and profibrotic gene expression.

To further explore the therapeutic potential of itaconate in IPF, human lung fibroblasts from IPF patients were treated with exogenous itaconate. This resulted in decreased maximal respiration, decreased spare respiratory capacity, limited proliferation, decreased wound closure, and decreased expression of *FN1* (encoding fibronectin-1) and *IL1B*. Underivatized itaconate was also administered therapeutically through inhalation to mice after bleomycin injury. Inhaled itaconate attenuated fibrosis with profibrotic factors and Ashcroft scoring being significantly reduced while dynamic resistance, elastance, and compliance were restored, indicating that inhaled itaconate could potentially be used therapeutically for patients with IPF, a disease currently with very few approved therapeutic options and a median survival of 3 years after diagnosis.

## Concluding remarks

Itaconate has emerged as a fascinating example of how metabolites act as signaling molecules in host defense and inflammation. The phenotypes observed in mice lacking *Irg1* and the therapeutic efficacy of itaconate and derivatives in vivo suggest that itaconate-related therapeutics could have an impact in treating infection and inflammatory diseases.

DMF, which is approved for the treatment of multiple sclerosis and psoriasis, is structurally similar to itaconate. As such, it also modifies cysteines, and it shares common mechanisms of action such as the activation of Nrf2, inhibition of GAPDH, and the targeting of GSDMD (9, 83, 108), highlighting the potential of itaconate or its derivatives.

A better understanding of the biology of itaconate will help inform us of how metabolic reprogramming orchestrates macrophage function and may reveal additional targets. Several interesting questions remain, including whether there is a receptor for itaconate, whether there are cellular export mechanisms, and how particular cysteine modifications induced by itaconate consequentially modulate protein function. We have much to learn about this fascinating Krebs cycle–derived metabolite, and information learned will help in the effort to explore its therapeutic potential.

## Figures and Tables

**Figure 1:**
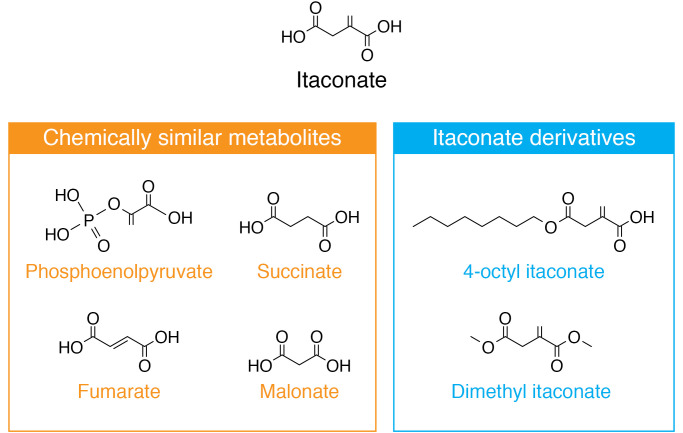
The structures of itaconate, similar metabolites, and its derivatives. Itaconate is a five-carbon dicarboxylic acid with an α,β-unsaturated alkene, making it mildly electrophilic. Structurally, itaconate is similar to several metabolites, including succinate, malonate, phosphoenolpyruvate, and fumarate. For instance, through structural similarity to succinate and malonate, itaconate can competitively inhibit succinate dehydrogenase and prevent the oxidation of succinate to fumarate (53, 54). Similarly, the alkene group allows itaconate to act as a Michael acceptor and react with cysteine residues in a similar manner to fumarate (11). Commonly used derivatives of itaconate include 4-octyl itaconate (OI) and dimethyl itaconate (DI), which are useful because of their high membrane permeability. While DI is not metabolized to itaconate (109), there is evidence that OI may be converted into itaconate intracellularly by esterases (11, 77, 78).

**Table 1:**
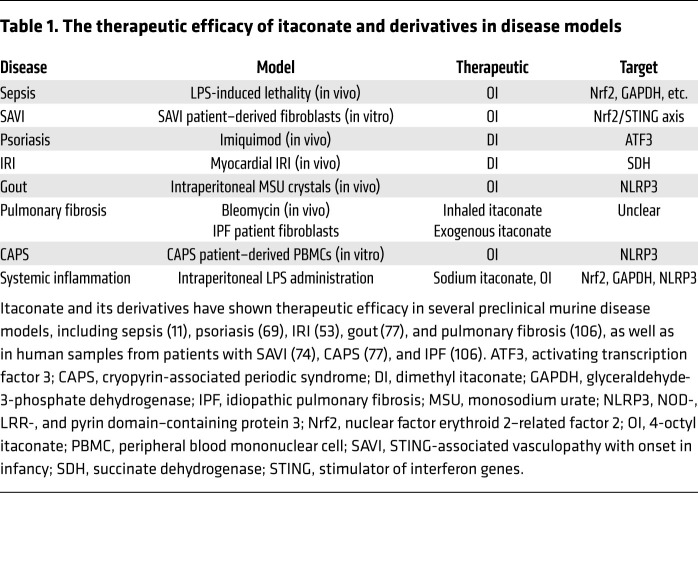
The therapeutic efficacy of itaconate and derivatives in disease models

## Version 1 01/18/2022

Electronic publication

## References

[1] Lunt SY, Vander Heiden MG (2011). Aerobic glycolysis: meeting the metabolic requirements of cell proliferation. Annu Rev Cell Dev Biol.

[2] Lauterbach MA (2019). Toll-like receptor signaling rewires macrophage metabolism and promotes histone acetylation via ATP-citrate lyase. Immunity.

[3] Seim GL (2019). Two-stage metabolic remodelling in macrophages in response to lipopolysaccharide and interferon-γ stimulation. Nat Metab.

[4] Ko JH (2020). BCAT1 affects mitochondrial metabolism independently of leucine transamination in activated human macrophages. J Cell Sci.

[5] Palmieri EM (2020). Nitric oxide orchestrates metabolic rewiring in M1 macrophages by targeting aconitase 2 and pyruvate dehydrogenase. Nat Commun.

[6] O’Neill LAJ (2015). A broken krebs cycle in macrophages. Immunity.

[7] Jha AK (2015). Network integration of parallel metabolic and transcriptional data reveals metabolic modules that regulate macrophage polarization. Immunity.

[8] Tannahill GM (2013). Succinate is an inflammatory signal that induces IL-1β through HIF-1α. Nature.

[9] Humphries F (2020). Succination inactivates gasdermin D and blocks pyroptosis. Science.

[10] Zasłona Z, O’Neill LAJ (2020). Cytokine-like roles for metabolites in immunity. Mol Cell.

[11] Mills EL (2018). Itaconate is an anti-inflammatory metabolite that activates Nrf2 via alkylation of KEAP1. Nature.

[12] Strelko CL (2011). Itaconic acid is a mammalian metabolite induced during macrophage activation. J Am Chem Soc.

[13] Shin JH (2011). 1H NMR-based metabolomic profiling in mice infected with mycobacterium tuberculosis. J Proteome Res.

[14] Michelucci A (2013). Immune-responsive gene 1 protein links metabolism to immunity by catalyzing itaconic acid production. Proc Natl Acad Sci U S A.

[15] Lee CGL (1995). Cloning and analysis of gene regulation of a novel LPS-inducible cDNA. Immunogenetics.

[16] O’Neill LAJ, Artyomov MN (2019). Itaconate: the poster child of metabolic reprogramming in macrophage function. Nat Rev Immunol.

[17] Swain A (2020). Comparative evaluation of itaconate and its derivatives reveals divergent inflammasome and type I interferon regulation in macrophages. Nat Metab.

[18] Pereira M (2019). Acute iron deprivation reprograms human macrophage metabolism and reduces inflammation in vivo. Cell Rep.

[19] McFadden BA, Purohit S (1977). Itaconate, an isocitrate lyase-directed inhibitor in Pseudomonas indigofera. J Bacteriol.

[20] Nguyen TV (2019). Itaconic acid inhibits growth of a pathogenic marine Vibrio strain: a metabolomics approach. Sci Rep.

[21] Williams JO (1971). Mechanism of action of isocitrate lyase from Pseudomonas indigofera. Biochemistry.

[22] McKinney JD (2000). Persistence of Mycobacterium tuberculosis in macrophages and mice requires the glyoxylate shunt enzyme isocitrate lyase. Nature.

[23] Muñoz-Elías EJ, McKinney JD (2005). Mycobacterium tuberculosis isocitrate lyases 1 and 2 are jointly required for in vivo growth and virulence. Nat Med.

[24] Lorenz MC, Fink GR (2002). Life and death in a macrophage: role of the glyoxylate cycle in virulence. Eukaryot Cell.

[25] Hillier S, Charnetzky WT (1981). Glyoxylate bypass enzymes in Yersinia species and multiple forms of isocitrate lyase in Yersinia pestis. J Bacteriol.

[26] Price JV (2019). IRG1 and inducible nitric oxide synthase act redundantly with other interferon-gamma-induced factors to restrict intracellular replication of legionella pneumophila. mBio.

[27] Naujoks J (2016). IFNs modify the proteome of legionella-containing vacuoles and restrict infection via IRG1-derived itaconic acid. PLOS Pathog.

[28] Ruetz M (2019). Itaconyl-CoA forms a stable biradical in methylmalonyl-CoA mutase and derails its activity and repair. Science.

[29] Berg IA (2002). Inhibition of acetate and propionate assimilation by itaconate via propionyl-CoA carboxylase in isocitrate lyase-negative purple bacterium Rhodospirillum rubrum. FEMS Microbiol Lett.

[30] Shen H (2017). The human knockout gene CLYBL connects itaconate to vitamin B12. Cell.

[31] Savvi S (2008). Functional characterization of a vitamin B12-dependent methylmalonyl pathway in Mycobacterium tuberculosis: implications for propionate metabolism during growth on fatty acids. J Bacteriol.

[32] Nair S (2018). Irg1 expression in myeloid cells prevents immunopathology during M. tuberculosis infection. J Exp Med.

[33] Chen M (2020). Itaconate is an effector of a Rab GTPase cell-autonomous host defense pathway against Salmonella. Science.

[34] Spanò S (2016). A bacterial pathogen targets a host Rab-family GTPase defense pathway with a GAP. Cell Host Microbe.

[35] Spanò S (2011). Proteolytic targeting of Rab29 by an effector protein distinguishes the intracellular compartments of human-adapted and broad-host Salmonella. Proc Natl Acad Sci U S A.

[36] Cooper RA (1965). The utilization of aconate and itaconate by micrococcus sp. Biochem J.

[37] Martin WR (1961). Noninductive metabolism of itaconic acid by Pseudomonas and Salmonella species. J Bacteriol.

[38] Cooper RA, Kornberg HL (1964). The utilization of itaconate by Pseudomonas sp. Biochem J.

[39] Sasikaran J (2014). Bacterial itaconate degradation promotes pathogenicity. Nat Chem Biol.

[40] Wang H (2019). An essential bifunctional enzyme in Mycobacterium tuberculosis for itaconate dissimilation and leucine catabolism. Proc Natl Acad Sci U S A.

[41] Papathanassiu AE (2017). BCAT1 controls metabolic reprogramming in activated human macrophages and is associated with inflammatory diseases. Nat Commun.

[42] Riquelme SA (2019). CFTR-PTEN-dependent mitochondrial metabolic dysfunction promotes Pseudomonas aeruginosa airway infection. Sci Transl Med.

[43] Riquelme SA (2020). Pseudomonas aeruginosa utilizes host-derived itaconate to redirect its metabolism to promote biofilm formation. Cell Metab.

[44] Juarez P (2017). Toxic electrophiles induce expression of the multidrug efflux pump MexEF-OprN in pseudomonas aeruginosa through a novel transcriptional regulator, CmrA. Antimicrob Agents Chemother.

[45] Wongsaroj L (2018). Pseudomonas aeruginosa glutathione biosynthesis genes play multiple roles in stress protection, bacterial virulence and biofilm formation. PLoS One.

[46] Maurice NM (2018). Pseudomonas aeruginosa biofilms: host response and clinical implications in lung infections. Am J Respir Cell Mol Biol.

[47] Tomlinson KL (2021). Staphylococcus aureus induces an itaconate-dominated immunometabolic response that drives biofilm formation. Nat Commun.

[48] Daniels BP (2019). The nucleotide sensor ZBP1 and kinase RIPK3 induce the enzyme IRG1 to promote an antiviral metabolic state in neurons. Immunity.

[49] Olagnier D (2020). SARS-CoV2-mediated suppression of NRF2-signaling reveals potent antiviral and anti-inflammatory activity of 4-octyl-itaconate and dimethyl fumarate. Nat Commun.

[50] Singh S (2021). Integrative metabolomics and transcriptomics identifies itaconate as an adjunct therapy to treat ocular bacterial infection. Cell Reports Med.

[51] Chen F (2019). Crystal structure of cis-aconitate decarboxylase reveals the impact of naturally occurring human mutations on itaconate synthesis. Proc Natl Acad Sci U S A.

[52] Mills EL (2016). Succinate dehydrogenase supports metabolic repurposing of mitochondria to drive inflammatory macrophages. Cell.

[53] Lampropoulou V (2016). Itaconate links inhibition of succinate dehydrogenase with macrophage metabolic remodeling and regulation of inflammation. Cell Metab.

[54] Cordes T (2016). Immunoresponsive gene 1 and itaconate inhibit succinate dehydrogenase to modulate intracellular succinate levels. J Biol Chem.

[55] Booth AN (1952). The inhibitory effects of itaconic acid in vitro and in vivo. J Biol Chem.

[56] Ackermann WW, Potter VR (1949). Enzyme inhibition in relation to chemotherapy. Proc Soc Exp Biol Med.

[57] Dervartanian DV, Veeger C (1964). Studies on succinate dehydrogenase. I. Spectral properties of the purified enzyme and formation of enzyme-competitive inhibitor complexes. Biochim Biophys Acta.

[58] Chandel NS (1998). Mitochondrial reactive oxygen species trigger hypoxia. Proc Natl Acad Sci U S A.

[59] McGettrick AF, O’Neill LAJ (2020). The role of HIF in immunity and inflammation. Cell Metab.

[60] Cummins EP (2016). The role of HIF in immunity and inflammation. Mol Aspects Med.

[61] Bauernfeind F (2011). Inflammasomes: current understanding and open questions. Cell Mol Life Sci.

[62] Domínguez-Andrés J (2019). The itaconate pathway is a central regulatory node linking innate immune tolerance and trained immunity. Cell Metab.

[63] Kobayashi EH (2016). Nrf2 suppresses macrophage inflammatory response by blocking proinflammatory cytokine transcription. Nat Commun.

[64] Itoh K (1999). Keap1 represses nuclear activation of antioxidant responsive elements by Nrf2 through binding to the amino-terminal Neh2 domain. Genes Dev.

[65] Campbell NK (2021). Regulation of inflammation by the antioxidant haem oxygenase 1. Nat Rev Immunol.

[66] Kumar H (2014). Natural product-derived pharmacological modulators of Nrf2/ARE pathway for chronic diseases. Nat Prod Rep.

[67] Dinkova-Kostova AT (2002). Direct evidence that sulfhydryl groups of Keap1 are the sensors regulating induction of phase 2 enzymes that protect against carcinogens and oxidants. Proc Natl Acad Sci U S A.

[68] Itoh K (1997). An Nrf2/small Maf heterodimer mediates the induction of phase II detoxifying enzyme genes through antioxidant response elements. Biochem Biophys Res Commun.

[69] Bambouskova M (2018). Electrophilic properties of itaconate and derivatives regulate the IκBζ–ATF3 inflammatory axis. Nature.

[70] Tang C (2018). 4-Octyl itaconate activates Nrf2 signaling to inhibit pro-inflammatory cytokine production in peripheral blood mononuclear cells of systemic lupus erythematosus patients. Cell Physiol Biochem.

[71] Cifani P (2021). Discovery of protein modifications using differential tandem mass spectrometry proteomics. J Proteome Res.

[72] Sun KA (2020). Endogenous itaconate is not required for particulate matter-induced NRF2 expression or inflammatory response. Elife.

[73] Bambouskova M (2021). Itaconate confers tolerance to late NLRP3 inflammasome activation. Cell Rep.

[74] Olagnier D (2018). Nrf2 negatively regulates STING indicating a link between antiviral sensing and metabolic reprogramming. Nat Commun.

[75] Qin W (2020). Chemoproteomic profiling of itaconation by bioorthogonal probes in inflammatory macrophages. J Am Chem Soc.

[76] Qin W (2019). S-glycosylation-based cysteine profiling reveals regulation of glycolysis by itaconate. Nat Chem Biol.

[77] Hooftman A (2020). The immunomodulatory metabolite itaconate modifies NLRP3 and inhibits inflammasome activation. Cell Metab.

[78] Liao ST (2019). 4-Octyl itaconate inhibits aerobic glycolysis by targeting GAPDH to exert anti-inflammatory effects. Nat Commun.

[79] Labzin LI (2015). ATF3 is a key regulator of macrophage IFN responses. J Immunol.

[80] Kelly B, O’Neill LAJ (2015). Metabolic reprogramming in macrophages and dendritic cells in innate immunity. Cell Res.

[81] Van Schaftingen E (1981). Inactivation of phosphofructokinase 2 by cyclic AMP — dependent protein kinase. Biochem Biophys Res Commun.

[82] Sakai A (2004). Itaconate reduces visceral fat by inhibiting fructose 2,6-bisphosphate synthesis in rat liver. Nutrition.

[83] Kornberg MD (2018). Dimethyl fumarate targets GAPDH and aerobic glycolysis to modulate immunity. Science.

[84] Ternette N (2013). Inhibition of mitochondrial aconitase by succination in fumarate hydratase deficiency. Cell Rep.

[85] McNab F (2015). Type I interferons in infectious disease. Nat Rev Immunol.

[86] Swanson KV (2019). The NLRP3 inflammasome: molecular activation and regulation to therapeutics. Nat Rev Immunol.

[87] Zhao C (2014). Nuclear factor E2-related factor-2 (Nrf2) is required for NLRP3 and AIM2 inflammasome activation. J Biol Chem.

[88] Chouchani ET (2014). Ischaemic accumulation of succinate controls reperfusion injury through mitochondrial ROS. Nature.

[89] Kula-Alwar D (2019). Targeting succinate metabolism in ischemia/reperfusion injury. Circulation.

[90] Cordes T (2020). Itaconate modulates tricarboxylic acid and redox metabolism to mitigate reperfusion injury. Mol Metab.

[91] Thimmulappa RK (2006). Nrf2 is a critical regulator of the innate immune response and survival during experimental sepsis. J Clin Invest.

[92] Okuma A (2013). Enhanced apoptosis by disruption of the STAT3-IκB-ζ signaling pathway in epithelial cells induces Sjögren’s syndrome-like autoimmune disease Immunity.

[93] Muromoto R (2016). IL-17A plays a central role in the expression of psoriasis signature genes through the induction of IκB-ζ in keratinocytes. Int Immunol.

[94] Johansen C (2015). IκBζ is a key driver in the development of psoriasis. Proc Natl Acad Sci U S A.

[95] Tsoi LC (2015). Enhanced meta-analysis and replication studies identify five new psoriasis susceptibility loci. Nat Commun.

[96] McGinley AM (2020). Interleukin-17A serves a priming role in autoimmunity by recruiting IL-1β-producing myeloid cells that promote pathogenic T cells. Immunity.

[97] Coll RC (2015). A small-molecule inhibitor of the NLRP3 inflammasome for the treatment of inflammatory diseases. Nat Med.

[98] Mangan MSJ (2018). Targeting the NLRP3 inflammasome in inflammatory diseases. Nat Rev Drug Discov.

[99] Duewell P (2010). NLRP3 inflammasomes are required for atherogenesis and activated by cholesterol crystals. Nature.

[100] Ising C (2019). NLRP3 inflammasome activation drives tau pathology. Nature.

[101] von Herrmann KM (2018). *NLRP3* expression in mesencephalic neurons and characterization of a rare NLRP3 polymorphism associated with decreased risk of Parkinson’s disease. NPJ Park Dis.

[102] Mortimer L (2016). NLRP3 inflammasome inhibition is disrupted in a group of auto-inflammatory disease CAPS mutations. Nat Immunol.

[103] Malhotra S (2020). NLRP3 inflammasome as prognostic factor and therapeutic target in primary progressive multiple sclerosis patients. Brain.

[104] Martinon F (2006). Gout-associated uric acid crystals activate the NALP3 inflammasome. Nature.

[105] Hoffman HM (2001). Mutation of a new gene encoding a putative pyrin-like protein causes familial cold autoinflammatory syndrome and Muckle-Wells syndrome. Nat Genet.

[106] Ogger PP (2020). Itaconate controls the severity of pulmonary fibrosis. Sci Immunol.

[107] Ashcroft T (1988). Simple method of estimating severity of pulmonary fibrosis on a numerical scale. J Clin Pathol.

[108] Linker RA (2011). Fumaric acid esters exert neuroprotective effects in neuroinflammation via activation of the Nrf2 antioxidant pathway. Brain.

[109] ElAzzouny M (2017). Dimethyl itaconate is not metabolized into itaconate intracellularly. J Biol Chem.

